# Adapting to the Host: Physiological Responses, Cell Wall Remodeling, and Persistence in Non-*albicans Candida* Species

**DOI:** 10.3390/jof12100791

**Published:** 2026-10-21

**Authors:** Manuela Gómez-Gaviria, Uriel Ramírez-Sotelo, Héctor M. Mora-Montes

**Affiliations:** Departamento de Biología, División de Ciencias Naturales y Exactas, Campus Guanajuato, Universidad de Guanajuato, Noria Alta s/n, col. Noria Alta, Guanajuato C.P. 36050, Guanajuato, Mexico; m.gomezgaviria@ugto.mx (M.G.-G.); u.ramirezsotelo@ugto.mx (U.R.-S.)

**Keywords:** non-*albicans Candida*, host adaptation, physiological adaptation, cell wall remodeling, fungal persistence, antifungal tolerance, host–pathogen interactions

## Abstract

Non-*albicans Candida* (NAC) species have emerged as clinically important opportunistic pathogens with distinct ecological, physiological, and pathogenic traits. Successful colonization and infection require these yeasts to survive within heterogeneous and continuously changing host microenvironments, where they encounter multiple and often simultaneous challenges. This review comparatively examines the adaptive responses of *Nakaseomyces glabratus*, *Candida parapsilosis*, *Candida tropicalis*, *Candida auris*, and *Pichia kudriavzevii* to major host-derived pressures, including thermal stress, nutrient restriction, fluctuations in oxygen availability and pH, and oxidative and nitrosative stress. Although exposed to similar environmental constraints, these species differ in how they integrate responses involving metabolic reprogramming, stress response mechanisms, cellular homeostasis, and structural modifications. Particular attention is given to cell wall remodeling as a dynamic interface linking environmental adaptation with adhesion, biofilm formation, immune recognition, and antifungal susceptibility. We further discuss how sustained adaptive responses may contribute to persistence and antifungal tolerance, thereby promoting fungal survival and complicating therapeutic eradication. Collectively, the pathogenic success of NAC species extends beyond classical virulence factors and depends on their capacity to dynamically adjust their physiology to host-imposed constraints, with both shared and species-specific strategies ultimately favoring survival and persistence during infection across diverse host niches under conditions of sustained therapeutic pressure.

## 1. Introduction

Human mycoses encompass a broad spectrum of diseases, ranging from superficial and mucocutaneous infections to life-threatening invasive and systemic conditions, whose incidence and clinical relevance have increased alongside the expansion of susceptible populations and the growing complexity of modern medical care [1,2]. The impact of these infections is further compounded by challenges in achieving early diagnosis, the limited number of available antifungal drug classes, and the increasing occurrence of species and isolates with reduced susceptibility to current therapies [3,4,5]. Among the major causative agents of invasive mycoses, yeasts of the genus *Candida* are particularly important because of their ability to transition from asymptomatic colonization of different host niches to the establishment of deep-seated and disseminated infections [6,7]. This transition represents a considerable biological challenge, as fungal cells must maintain their viability while encountering host microenvironments with markedly different physicochemical, nutritional, and immunological conditions. Therefore, the success of *Candida* as an opportunistic pathogen depends not only on its ability to colonize and invade the host, but also on its capacity to dynamically adjust its physiology to the adverse and changing conditions encountered throughout infection progression [8,9,10,11].

Over the past decades, the epidemiology of candidiasis has undergone a significant shift, characterized by an increasing proportion of infections caused by non-*albicans Candida* (NAC) species, whose prevalence varies considerably according to geographic region, patient population, and clinical practices [10,11,12]. Among these, *Nakaseomyces glabratus*, *Candida parapsilosis*, *Candida tropicalis*, *Candidozyma auris*, and *Pichia kudriavzevii* have gained particular clinical relevance and display distinct biological and epidemiological features that complicate the management of the infections they cause [12,13,14,15]. These species differ in their reservoirs, colonization and transmission capacities, tropism for specific host niches, and antifungal susceptibility profiles, resulting in distinct patterns of infection and therapeutic challenges [6,11,16]. Some exhibit intrinsically reduced susceptibility to particular antifungal classes, whereas others show a remarkable ability to persist on medical devices, form biofilms, survive in healthcare environments, or acquire resistance during antifungal treatment [9,10,17]. Collectively, the increasing clinical prominence of NAC species highlights that the pathogenic success of these yeasts does not rely on a single strategy but can arise from distinct biological solutions that enable phylogenetically and ecologically diverse species to colonize the host, persist during infection, and withstand therapeutic pressure [11,18].

Traditionally, the pathogenicity of *Candida* species has been explained by a repertoire of virulence factors that promote host colonization, tissue invasion, and damage, including adhesion, biofilm formation, morphological plasticity, secretion of hydrolytic enzymes, and immune evasion mechanisms [19,20,21]. However, the presence of these attributes alone does not fully explain the ability of fungal populations to maintain viability and fitness throughout the different stages of infection [19]. Establishment and persistence within the host require fungal cells to continuously respond to environmental changes by adjusting processes such as energy metabolism, cellular homeostasis, stress responses, and cell surface organization [19]. In this context, pathogenicity can be understood not merely as the result of a fixed repertoire of virulence factors, but rather as a dynamic property in which the capacity to adjust fungal physiology in response to environmental conditions plays a key role in microbial survival and persistence [19,22,23].

During infection, fungal cells transition between host niches that differ considerably in their physicochemical, nutritional, and immunological characteristics [12]. Colonization of epithelial surfaces, tissue invasion, dissemination through the bloodstream, biofilm establishment, and interactions with phagocytic cells expose *Candida* populations to distinct biological environments that may further change as infection progresses [6,15]. These transitions involve variations in temperature, nutrient and micronutrient availability, oxygen tension, and pH, as well as exposure to reactive oxygen and nitrogen species and other effectors of the host immune response [11,21,24]. Rather than acting independently, these conditions may occur simultaneously, imposing physiological constraints that require fungal cells to coordinate multiple responses to maintain cellular homeostasis. Thus, the spatial and temporal heterogeneity of the host makes infection a dynamic process in which the ability to sense environmental changes and integrate adaptive responses is essential for maintaining fungal fitness across the different niches encountered during colonization and disease progression [7,11,21].

In response to this diversity of conditions, *Candida* species deploy adaptive responses that involve the coordinated reorganization of multiple cellular processes. These adjustments may include the reprogramming of metabolism and nutrient acquisition pathways, activation of stress response mechanisms, and maintenance of redox and protein homeostasis, as well as changes in the composition and organization of the cell membrane and cell wall [25,26]. Such responses not only enable fungal cells to counteract the disturbances imposed by adverse environments but can also modify properties directly involved in host–pathogen interactions, including morphology, adhesion, biofilm formation, and recognition by the immune system [26]. However, the way in which these responses are prioritized and integrated varies among NAC species, reflecting their distinct physiological and ecological characteristics. Thus, similar environmental pressures may lead to distinct adaptive strategies while converging on a common outcome: preserving cellular fitness and promoting fungal survival and persistence within the host [12,27].

In this context, the present review comparatively examines how major host-derived pressures shape the adaptive responses of clinically relevant NAC species, with particular emphasis on *N. glabratus*, *C. parapsilosis*, *C. tropicalis*, *C. auris*, and *P. kudriavzevii*. We examine the mechanisms employed to cope with thermal stress, nutrient restriction, and fluctuations in oxygen availability and pH, as well as oxidative and nitrosative stress, highlighting both shared strategies and species-specific features. We also discuss cell wall remodeling as a dynamic interface linking adaptation to environmental conditions with fungal–host interactions, and explore how the sustained integration of these responses may contribute to persistence and antifungal tolerance. Through this comparative perspective, we aim to provide an integrative framework for understanding how species with distinct biological and ecological characteristics can respond to common host-associated challenges and converge on phenotypes that promote survival and pathogenic success during infection.

This narrative review was developed through a literature search conducted between June and September 2026 using PubMed, Scopus, and Google Scholar. Relevant publications were identified using combinations of species names and keywords related to host adaptation, physiological stress responses, cell wall remodeling, immune recognition, antifungal tolerance, and persistence. Priority was given to original experimental studies providing mechanistic insights into the adaptive responses of non-*albicans Candida* species, particularly those investigating conditions relevant to host colonization and infection. Review articles were also consulted to provide broader conceptual and clinical context. Given the narrative nature of this review, publications were selected based on their relevance to the topics addressed rather than through a formal systematic screening process.

## 2. Evolution Under Host-Derived Selective Pressures

The infection process caused by NAC species is not a static biological event but rather a dynamic process of continuous evolution, in which fungal populations are constantly exposed to multiple host-derived selective pressures [12,19]. Unlike the relatively stable conditions of laboratory culture media, the host environment represents a mosaic of heterogeneous microenvironments that differ in temperature, nutrient availability, oxygen tension, pH, oxidative stress, and exposure to immune cells and soluble immune mediators [11,12]. Moreover, the initiation of antifungal therapy introduces an additional selective pressure that further reshapes the composition of the infecting population [28].

From an evolutionary perspective, each of these factors acts as a selective filter that continuously reshapes the genetic and phenotypic composition of *Candida* populations [11]. Body temperature, nutrient restriction, limitation of iron and other essential metals, fluctuations in pH and oxygen availability, the production of reactive oxygen and nitrogen species, immune recognition, and exposure to antifungal agents should not be regarded as independent challenges but rather as interconnected evolutionary forces acting simultaneously throughout infection [29,30,31]. Consequently, successful adaptation depends on the ability of fungal cells to integrate physiological, metabolic, and structural responses that collectively ensure survival under these continuously changing conditions [27].

### 2.1. Temperature and Thermal Stress

Temperature represents one of the earliest and most pervasive host-derived selective pressures encountered by fungal pathogens during the establishment of infection [32,33]. The transition from environmental reservoirs or colonized body surfaces to the human host exposes fungal cells to a sustained increase in temperature, imposing profound challenges to protein stability, plasma membrane fluidity, metabolic activity, mitochondrial function, and cell wall integrity [34,35,36]. Consequently, thermotolerance should not be viewed merely as a physiological requirement for growth at 37 °C, but rather as an adaptive trait shaped by natural selection that continues to be refined under host-associated conditions [35]. In this context, NAC species have evolved both highly conserved stress-response mechanisms and species-specific adaptive strategies that enable them to maintain cellular homeostasis and persist under thermal stress (Figure 1) [12]. Although all NAC species are capable of proliferating at human body temperature, they differ considerably in their degree of thermotolerance and in the molecular mechanisms they employ to cope with heat stress [35,37,38]. These differences likely reflect the distinct evolutionary histories and ecological niches of each species, underscoring that thermal adaptation does not rely on a single conserved mechanism but rather on the coordinated integration of multiple physiological, metabolic, and regulatory responses [34].

Among NAC species, *N. glabratus* exhibits a remarkable intrinsic tolerance to elevated temperatures and other environmental stresses, a characteristic that likely contributes to its ability to colonize diverse host niches and persist during infection [39,40]. Its optimal growth temperature is approximately 37 °C, and exposure to heat stress triggers a coordinated adaptive response involving proteostasis, cellular signaling, cell wall remodeling, and metabolic reprogramming [41]. Central components of this response include mitogen-activated protein kinase (MAPK) signaling pathways, particularly the high-osmolarity glycerol (HOG) and cell wall integrity (CWI) pathways, together with the general stress-response regulators Msn2 and Msn4 [42,43]. Of particular importance is the Sho1-dependent branch of the HOG pathway, whose disruption compromises growth at 42 °C, demonstrating its direct contribution to thermotolerance in *N. glabratus* [42,44,45].

The thermal response of *N. glabratus* is part of a broader adaptive program rather than a heat-specific reaction. Transcriptomic analyses performed under thermal, osmotic, oxidative, genotoxic, and cell wall stresses have shown that nearly 75% of the genome undergoes transcriptional changes in response to these conditions, whereas approximately one-quarter of all genes participate in a conserved adaptive response shared among multiple stressors [39,42,46]. This common stress program is characterized by the upregulation of translation-associated functions and the downregulation of genes involved in mitochondrial activity, suggesting a redistribution of cellular resources toward maintaining viability and repairing stress-induced damage [46]. Moreover, the identification of numerous putative transcriptional regulators supports the existence of an integrated regulatory network that enables *N. glabratus* to respond simultaneously to the diverse environmental challenges encountered within the host [43].

The adaptive potential of *N. glabratus* under heat stress has also been demonstrated through experimental evolution studies [39]. Repeated exposure to progressively increasing temperatures ranging from 47 to 50 °C resulted in the selection of populations displaying enhanced thermotolerance together with cross-protection against oxidative agents, organic acids, ethanol, and other environmental stressors [39]. Remarkably, all evolved populations acquired mutations in *CgSTE11*, which encodes a MAPKKK involved in coordinating multiple signaling pathways [47]. The frequency of these variants rapidly increased until fixation within the evolving populations, and reconstruction of the mutations in the parental genetic background confirmed that they conferred not only increased thermotolerance but also cross-tolerance to hydrogen peroxide, ethanol, and acetic acid. These findings provide direct evidence that temperature can function as an evolutionary filter capable of selecting genetic changes that simultaneously enhance resistance to multiple environmental challenges [39].

The adaptive function of CgSte11 appears to depend, at least in part, on Hsp90 activity and on the coordinated interaction between the HOG and CWI signaling pathways [39]. Heat-adapted variants exhibited marked hypersensitivity to elevated temperatures following pharmacological inhibition of Hsp90, highlighting the essential role of this molecular chaperone in stabilizing key signaling components required for thermal adaptation [48]. Collectively, these findings indicate that thermotolerance in *N. glabratus* emerges from the integration of a conserved transcriptional stress response, coordinated signaling networks, and the selection of genetic variants with pleiotropic adaptive effects [40,46]. Thus, heat should not be viewed merely as a physiological challenge for this species but rather as an evolutionary force capable of shaping fungal populations by selecting phenotypes with enhanced fitness under the dynamic conditions encountered within the host.

In contrast to the broad environmental stress tolerance exhibited by *N. glabratus*, thermal adaptation in *C. parapsilosis* appears to rely largely on its ability to modify cell surface properties and adjust its adhesive behavior in response to temperature changes [34,49]. This characteristic is particularly relevant for a species closely associated with skin colonization, transmission via the hands of healthcare workers, and biofilm formation on intravascular catheters and other medical devices [50,51]. Studies using clinical isolates have demonstrated that growth at 22 and 37 °C alters cell surface hydrophobicity, surface free energy, and adhesion to polystyrene; however, both the magnitude and direction of these changes vary considerably among strains [34]. While some isolates maintain a highly hydrophobic phenotype at both temperatures, others remain predominantly hydrophilic, highlighting substantial intraspecific heterogeneity in the way temperature remodels the fungal interface with both biotic and abiotic surfaces [49].

This strain-dependent response is also evident during the early stages of adhesion and biofilm development [34]. In an analysis of nosocomial isolates exposed to temperatures ranging from 31 to 43 °C, *C. parapsilosis* retained its ability to adhere and form biofilms at temperatures above normal body temperature, although biofilm biomass generally declined at temperatures exceeding 37 °C [34]. Notably, some isolates maintained substantial biofilm production up to 42 °C, whereas initial adhesion and subsequent biofilm growth displayed partially independent thermal responses. These findings indicate that temperature does not regulate all stages of biofilm development uniformly; instead, it may preserve or even promote the initial attachment to a surface while subsequently restricting biofilm proliferation and maturation [34]. From an adaptive perspective, this dissociation is particularly relevant, as febrile temperatures that temporarily limit growth may still allow specific variants to remain attached and resume biofilm development once environmental conditions become more favorable.

Overall, the available evidence suggests that, in *C. parapsilosis*, temperature functions not only as a physiological constraint but also as an environmental cue capable of remodeling traits directly associated with its success as a nosocomial pathogen. Temperature-dependent changes in cell surface hydrophobicity, adhesion, and biofilm formation may significantly influence its ability to colonize medical devices, persist on polymeric surfaces, and disseminate between hosts. Unlike *N. glabratus*, whose adaptation to heat relies primarily on a robust environmental stress response, and *C. parapsilosis*, in which temperature mainly modulates adhesion and biofilm formation, thermal stress in *C. tropicalis* acts as a central regulator of morphogenesis, biofilm architecture, and the expression of multiple virulence determinants [52]. Although this species proliferates efficiently at human body temperature, exposure to elevated temperatures triggers extensive physiological reprogramming involving metabolic adaptation, cell wall remodeling, and transitions between distinct morphological states, thereby promoting adaptation to diverse host niches [36,53]. Consequently, temperature not only determines the survival of *C. tropicalis* but also functions as an environmental signal that directly modulates its pathogenic behavior [54].

Several studies have shown that the heat stress response of *C. tropicalis* shares conserved adaptive mechanisms with other *Candida* species, including the activation of heat shock proteins, proteostasis networks, and pathways involved in maintaining cell wall integrity [36]. However, this response displays distinctive features owing to the close interplay between temperature, filamentation, and biofilm development [53]. Analyses of clinical isolates have demonstrated that both initial adhesion and biofilm growth respond differentially to temperatures ranging from 31 to 43 °C, with considerable strain-dependent variability [54,55]. While some isolates retain a high capacity for adhesion and biofilm formation even under febrile conditions, others exhibit a marked reduction in biofilm biomass, indicating that temperature acts as an important selective force favoring adaptive phenotypes during infection.

*C. auris* probably represents the most compelling example of how adaptation to thermal stress may have contributed to the emergence of a novel human fungal pathogen [56]. Unlike most members of the *Candida haemulonii* species complex, *C. auris* exhibits a remarkable ability to grow at temperatures up to 42 °C, a characteristic that has been widely associated with its adaptation to the mammalian host and its success as an emerging nosocomial pathogen [57,58]. This exceptional thermotolerance has led to the hypothesis that progressive adaptation to increasing environmental temperatures, potentially driven by climate change, acted as a selective force that enabled *C. auris* to overcome the mammalian thermal barrier and facilitate its transition from environmental reservoirs to the human host [56,58].

Adaptation to heat in *C. auris* relies on a complex signaling network integrating heat shock proteins, proteostasis mechanisms, and multiple stress-response pathways [59]. Among these, Hsp90 plays a pivotal role by stabilizing essential regulatory proteins and coordinating the cellular response to elevated temperatures, whereas the HOG, calcineurin, protein kinase A (PKA), and CWI pathways act in concert to preserve cellular homeostasis, remodel the cell surface, and promote survival under thermal stress [59,60]. Experimental disruption of these pathways markedly impairs growth at elevated temperatures, demonstrating that thermotolerance in *C. auris* is an actively regulated adaptive trait rather than a passive physiological property [57,60].

Beyond promoting cellular survival, temperature also modulates several traits directly associated with the pathogenicity of *C. auris*. Recent studies have shown that thermal stress induces coordinated changes in cell wall composition, biofilm organization, antifungal tolerance, and responses to multiple environmental challenges, highlighting the extensive crosstalk between heat adaptation pathways and the mechanisms underlying persistence and antifungal resistance [56,61]. Consequently, thermotolerance not only enhances the fitness of *C. auris* within the mammalian host but also promotes its persistence on hospital surfaces, colonization of human skin, and tolerance to the diverse stresses encountered during infection.

Unlike the species discussed above, the remarkable thermotolerance of *P. kudriavzevii* appears to be closely linked to its broad ecological distribution and its ability to thrive in industrial and fermentative environments exposed to elevated temperature [58,62,63]. This yeast has been exploited for decades in biotechnological processes because of its ability to grow efficiently at temperatures between 40 and 45 °C, tolerate thermal fluctuations, and maintain active fermentative metabolism under conditions that restrict the growth of many other yeasts. These characteristics suggest that thermotolerance represents an ecological adaptation acquired during the evolution of the species rather than a trait that evolved exclusively after its transition to the human host [63].

Transcriptomic and metabolomic studies further demonstrate that the response of *P. kudriavzevii* to thermal stress involves extensive physiological reprogramming, including the induction of heat shock proteins, activation of antioxidant defense systems, remodeling of energy metabolism, alterations in membrane lipid composition, and profound changes in carbon and amino acid metabolic pathways [57]. Together, these adaptive responses preserve cellular homeostasis, maintain protein stability, and minimize oxidative damage caused by elevated temperatures, thereby conferring remarkable resilience under adverse environmental conditions. Moreover, prior exposure to heat can induce cross-protection against other environmental stresses, illustrating the tight integration between thermal adaptation and broader cellular stress-response networks [58]. Beyond these physiological responses, temperature-associated changes in cellular morphology have also been described in *P. kudriavzevii*. Filamentous growth has been observed at 37 °C in RPMI medium, whereas blastoconidia and pseudohyphal forms have been reported under other cultivation conditions. These observations suggest that the morphological repertoire of this species may also respond to environmental conditions relevant to the mammalian host, although the specific contribution of temperature and other culture-dependent factors requires further investigation [64].

From an evolutionary perspective, the thermotolerance of *P. kudriavzevii* may represent a form of ecological preadaptation, whereby physiological traits associated with survival in environmental and fermentative niches could also facilitate growth under mammalian temperature conditions [35,58]. However, the mechanisms underlying thermal adaptation have been characterized predominantly in environmental or industrial contexts, and their contribution to fungal fitness during human infection remains poorly understood. Although the ability to grow at 37 °C and the occurrence of filamentous growth under host-relevant culture conditions support the physiological compatibility of this species with mammalian environments, these observations do not establish a direct relationship between thermotolerance, morphological adaptation, and pathogenicity. Further studies using clinical isolates and infection-relevant experimental models are therefore needed to determine whether these responses contribute to survival and persistence within the host [61].

### 2.2. Nutrient Restriction

After overcoming the thermal barrier and initiating host colonization, NAC species encounter a second major selective pressure: nutrient limitation [27]. Unlike the nutrient-rich culture media routinely used under laboratory conditions, host tissues represent highly restrictive environments in which access to carbon and nitrogen sources, iron, zinc, copper, and other essential micronutrients is tightly regulated [12,27]. Rather than being a passive consequence of the host environment, this nutrient deprivation constitutes an active defense mechanism known as nutritional immunity, whereby the host deliberately restricts the availability of essential nutrients to limit microbial proliferation. Consequently, the ability to sense fluctuations in nutrient availability, reprogram cellular metabolism, and activate specialized nutrient acquisition systems has emerged as a key determinant of the adaptation, persistence, and pathogenic success of *Candida* species during infection [65].

From an evolutionary perspective, nutrient limitation acts as a powerful selective force that promotes the expansion of variants with greater metabolic efficiency and enhanced physiological flexibility [66,67]. Adaptation to nutrient deprivation depends not only on the induction of high-affinity transporters and specialized metal acquisition systems but also on extensive metabolic reprogramming that enables fungal cells to exploit alternative carbon and nitrogen sources, redistribute energetic resources, and coordinate changes in cell wall composition, mitochondrial function, and stress-response networks [68]. As a result, the outcome of infection largely depends on the ability of the fungus to integrate nutritional cues with adaptive physiological responses that promote survival across the diverse microenvironments encountered within the host [66].

In *N. glabratus*, adaptation to nutrient limitation represents a central component of its ability to persist within the host, particularly during intracellular survival inside macrophages [69,70,71,72]. Unlike nutrient-rich laboratory media, the phagosome constitutes a highly restrictive compartment characterized by limited availability of carbon and nitrogen sources, vitamins, and essential micronutrients, while simultaneously being subjected to active mechanisms of nutritional immunity. To cope with these conditions, *N. glabratus* integrates cellular recycling pathways, high-affinity nutrient transport systems, and specialized regulatory networks that enable it to maintain viability and continue replicating within this hostile intracellular environment [73,74]. Autophagy plays a pivotal role in this process by recycling intracellular components under nutrient-deprived conditions, whereas chromatin remodeling and metabolic reprogramming contribute to the efficient redistribution of cellular resources following phagocytic internalization [70,71].

A particularly distinctive feature of *N. glabratus* is its auxotrophy for nicotinic acid and its dependence on exogenous NAD^+^ precursors [75]. Under niacin-limiting conditions, the fungus induces high-affinity transporters for NAD^+^ precursors through the Hst1–Rfm1–Sum1 repressor complex. This response enhances the uptake of nicotinic acid, nicotinamide, and other NAD^+^ precursors when their availability declines, thereby sustaining redox metabolism and numerous NAD^+^-dependent cellular processes [75]. The coordinated regulation of these transporters demonstrates that vitamin limitation is not merely a metabolic deficiency but also an environmental cue capable of activating adaptive programs that promote survival within nutritionally restrictive host niches [75].

Iron homeostasis represents another fundamental axis of this adaptive response. Compared with *C. albicans*, *N. glabratus* utilizes a more restricted repertoire of iron sources, efficiently acquiring ferritin-bound and non-protein-bound iron, particularly under acidic conditions, while displaying limited capacity to exploit transferrin, heme, or hemoglobin as iron sources [76,77,78]. Iron uptake relies primarily on the high-affinity reductive iron assimilation system composed of the iron permease Ftr1 and the multicopper ferroxidase Fet3. However, unlike most pathogenic fungi, *N. glabratus* does not require detectable cell surface ferric reductase activity for efficient iron acquisition [79,80]. Instead, it secretes a low-molecular-weight reducing compound capable of reducing extracellular ferric iron, thereby compensating for the absence of surface ferric reductases. This strategy represents a distinctive evolutionary solution for iron acquisition under the restrictive conditions imposed by the host [78].

The importance of iron limitation becomes particularly evident during macrophage interaction. Functional analyses of *N. glabratus* mutant libraries have identified genes involved in iron homeostasis, nutrient adaptation, cell wall biosynthesis, and stress resistance as essential determinants of intracellular survival [78]. Although carbon and nitrogen limitation exert relatively modest effects on the fitness of many mutants, iron restriction profoundly compromises fungal growth and survival, indicating that iron availability constitutes one of the major nutritional bottlenecks within the phagosome. Efficient iron acquisition and homeostasis, together with autophagy and metabolic reprogramming, enable specific subpopulations to tolerate the intraphagosomal environment, persist within macrophages, and evade immune-mediated clearance [78,81].

Iron homeostasis in *N. glabratus* is governed by a hybrid regulatory network that combines regulatory features conserved in *Saccharomyces cerevisiae* with mechanisms characteristic of pathogenic fungi [82]. The transcription factors Aft1 and Aft2 primarily activate genes required during iron starvation, whereas Yap5 and the CCAAT-binding complex coordinate iron storage, mitochondrial respiration, and adaptation to iron excess. This regulatory organization enables the fungus to finely balance iron uptake, utilization, and storage according to environmental availability, thereby preventing both iron deficiency and iron toxicity [68]. Collectively, these findings demonstrate that nutrient adaptation in *N. glabratus* relies on the tight integration of environmental sensing, transcriptional reprogramming, and metabolic redistribution, mechanisms that collectively promote survival within nutritionally restrictive host niches and contribute to fungal persistence during infection [68].

For *C. parapsilosis*, adaptation to nutrient limitation appears to be closely linked to iron acquisition and homeostasis, as iron is one of the micronutrients most severely restricted during infection as a consequence of host nutritional immunity [83]. Although iron acquisition systems in this species have been less extensively characterized than those of *C. albicans*, the available evidence demonstrates that iron homeostasis is a central determinant of both cellular metabolism and the expression of virulence-associated traits [84]. Consequently, iron availability not only influences fungal growth but also acts as an environmental signal capable of modulating adaptive processes directly associated with host colonization and persistence [83].

The reductive iron assimilation pathway represents one of the major mechanisms employed by *C. parapsilosis* to cope with iron limitation. A key component of this system is the multicopper ferroxidase Fet3, whose inactivation severely impairs high-affinity iron uptake and leads to profound alterations in fungal physiology [85]. *FET3*-deficient mutants exhibit impaired growth under iron-depleted conditions, a marked reduction in pseudohyphal formation, and a diminished capacity to develop mature biofilms [83]. These findings indicate that iron homeostasis is not merely a metabolic process but rather a regulatory hub that simultaneously coordinates nutrient acquisition, morphogenesis, and biofilm development, thereby integrating metabolism and virulence into a single adaptive program [83].

The importance of iron extends well beyond the regulation of individual morphological traits [83]. Transcriptomic analyses have shown that iron deprivation triggers extensive transcriptional reprogramming affecting pathways involved in energy metabolism, mitochondrial respiration, cofactor biosynthesis, and oxidative stress responses [83]. This metabolic reprogramming enables the redistribution of cellular resources while maintaining essential physiological functions under iron-limiting conditions, illustrating that adaptation to nutrient restriction involves a global reorganization of fungal physiology rather than the simple induction of high-affinity transport systems.

Beyond iron homeostasis, recent studies have revealed that *C. parapsilosis* possesses distinctive regulatory networks that enable adaptation to the availability of other essential nutrients. A notable example is sulfur metabolism, whose regulation differs substantially from that described in other *Candida* species [86]. The transcription factors Met4 and Met28 coordinate the expression of genes involved in sulfate assimilation and the biosynthesis of cysteine and methionine, thereby adjusting sulfur metabolism according to environmental nutrient availability [87]. These findings indicate that *C. parapsilosis* has evolved species-specific regulatory strategies to cope with nutrient limitation, reflecting an evolutionary trajectory distinct from that of other members of the genus [87].

In *C. tropicalis*, adaptation to nutrient limitation is primarily characterized by remarkable metabolic versatility, which enables this species to exploit a broad range of alternative carbon and nitrogen sources available across diverse host niches [88,89]. During infection, fungal cells encounter environments in which glucose and other readily assimilable nutrients become scarce owing to both competition with the resident microbiota and the host’s nutritional immunity mechanisms [88]. In response, *C. tropicalis* undergoes extensive metabolic rewiring to utilize alternative substrates while maintaining growth and energy production under nutrient-limiting conditions [54]. This metabolic adaptability is likely one of the major determinants underlying its success as a colonizer of multiple host tissues and mucosal surfaces [90].

Physiological studies have demonstrated that *C. tropicalis* can grow using a wide variety of carbohydrates, alcohols, organic acids, and nitrogen-containing compounds as sole carbon or nitrogen sources, reflecting a remarkable metabolic versatility compared with many other pathogenic yeasts [88,90]. This broad metabolic repertoire not only promotes survival in environmental and food-associated habitats but also enhances the ability of *C. tropicalis* to adapt to the heterogeneous microenvironments encountered during human infection, where nutrient availability continuously varies among tissues, body fluids, and biofilms [90]. Furthermore, the coordinated regulation of catabolic pathways enables the fungus to preferentially exploit the most energetically favorable carbon sources while retaining the capacity to rapidly switch to alternative substrates when preferred nutrients become depleted [91].

Adaptation to nutrient deprivation also involves profound physiological remodeling. Recent transcriptomic studies have shown that nutrient limitation induces coordinated changes in pathways associated with central carbon metabolism, mitochondrial respiration, amino acid biosynthesis, and oxidative stress responses, highlighting the close integration between nutrient availability and global metabolic regulation [53]. These adaptive responses promote the redistribution of energetic resources, preserve cellular homeostasis, and maintain essential physiological functions even under severe nutrient limitation.

The availability of different carbon sources alters biofilm architecture, cell wall composition, interactions with host immune cells, and antifungal tolerance, indicating that nutritional cues regulate not only metabolism but also the pathogenic phenotype of *C. tropicalis* [54]. From an evolutionary perspective, this ability to continuously reprogram cellular metabolism in response to nutrient availability provides a significant adaptive advantage, promoting colonization of multiple host niches, persistence during infection, and survival under the selective pressures imposed by the host nutritional environment.

In *C. auris*, adaptation to nutrient limitation represents one of the major factors underlying its remarkable ability to persist both within the host and in the hospital environment. Unlike most other *Candida* species, *C. auris* efficiently colonizes human skin, where it encounters limited availability of carbon, nitrogen, and essential micronutrients, together with high salt concentrations, fatty acids, and a variety of antimicrobial compounds [27,92]. To survive under these challenging conditions, the fungus exhibits remarkable metabolic plasticity, rapidly rewiring its biosynthetic pathways and adjusting nutrient utilization according to the physicochemical characteristics of each microenvironment [93].

Transcriptomic and metabolomic analyses have demonstrated that nutrient limitation induces extensive remodeling of central metabolism in *C. auris*, affecting pathways involved in carbon metabolism, amino acid biosynthesis, mitochondrial respiration, and energy production [94,95]. These metabolic changes are accompanied by coordinated remodeling of the cell wall, biofilm architecture, and oxidative stress responses, indicating that adaptation to nutrient deprivation involves an integrated physiological response that extends well beyond nutrient acquisition alone [96,97]. The tight coupling between metabolic regulation and cellular architecture enables *C. auris* to maintain homeostasis while colonizing nutrient-restricted niches exposed to multiple environmental stresses.

Another distinctive feature of *C. auris* is the close relationship between metabolism and antifungal tolerance. Several studies have shown that alterations in nutrient utilization and energy metabolism simultaneously influence cellular physiology, membrane lipid composition, and susceptibility to antifungal agents, thereby promoting persistence during prolonged antifungal therapy [94,96]. From an evolutionary perspective, this remarkable metabolic plasticity provides a broad adaptive landscape that facilitates the selection of phenotypes capable of surviving under nutrient-limited conditions, persisting in hospital environments, and rapidly adapting to both new ecological niches and the human host.

Finally, in *P. kudriavzevii*, the physiological responses to nutrient limitation remain poorly characterized in the context of human infection. Nevertheless, experimental evidence indicates that nutrient availability can influence growth behavior and morphological plasticity in this species. In particular, nutrient-limited conditions have been associated with changes in growth patterns, including pseudohyphal development and penetrative growth in agar [98]. These observations suggest that nutrient availability may act as an environmental cue influencing morphological adaptation under unfavorable conditions. However, because these findings were obtained using a food-associated isolate under laboratory conditions, their relevance to nutrient acquisition and survival within the host remains uncertain. Further studies involving clinical isolates and infection-relevant models are therefore required to determine whether nutrient-dependent morphological responses contribute to fungal adaptation and persistence during infection.

### 2.3. Fluctuations in pH and Oxygen

Following adaptation to nutrient limitation, NAC species encounter another major host-derived selective pressure: fluctuations in oxygen availability and environmental pH within the infection microenvironment [27,66]. Unlike the relatively homogeneous conditions provided by laboratory culture media, host niches exhibit pronounced physicochemical heterogeneity shaped by tissue type, inflammatory responses, cellular metabolism, and the activity of the resident microbiota. Consequently, throughout colonization and disease progression, fungal populations are continuously exposed to microenvironments that differ markedly in oxygen tension and acidity, including mucosal surfaces, biofilms, phagosomes, inflamed tissues, and necrotic lesions [12,66].

From an evolutionary perspective, both oxygen availability and environmental pH function as powerful selective forces capable of profoundly reshaping fungal physiology. Reduced oxygen tension influences mitochondrial respiration, energy production, lipid biosynthesis, and oxidative stress responses, whereas fluctuations in pH alter nutrient availability, ion homeostasis, cell wall architecture, and the activity of numerous enzymes involved in host–pathogen interactions [27,66]. Rather than acting as independent environmental cues, oxygen and pH are integrated through interconnected regulatory networks that coordinate transcriptional, metabolic, and structural responses to preserve cellular homeostasis under continuously changing host conditions [11].

The ability to sense these environmental changes and mount coordinated adaptive responses is a fundamental determinant of fungal fitness during infection. Although NAC species share conserved mechanisms that facilitate adaptation to hypoxia and pH fluctuations, accumulating evidence indicates that each species has evolved distinct physiological strategies that reflect its evolutionary history, preferred ecological niches, and pathogenic lifestyle [99]. These species-specific adaptations influence not only the efficiency with which individual species colonize different host tissues but also their capacity to persist during infection and withstand additional selective pressures, including host immunity and antifungal therapy [11].

In *N. glabratus*, adaptation to hypoxia appears to rely primarily on extensive remodeling of respiratory metabolism and on the ability to preserve energy homeostasis when mitochondrial activity becomes limited [100]. Reduced oxygen availability alters sterol biosynthesis, lipid metabolism, and cell wall composition, thereby preserving plasma membrane integrity and cellular viability under oxygen-restricted conditions. This adaptive strategy is consistent with the predominantly yeast-form lifestyle of *N. glabratus* and its remarkable ability to survive within macrophages, where it is simultaneously exposed to hypoxia, acidification, oxidative stress, and nutrient limitation [101,102]. Within these intracellular compartments, adaptation to acidic pH requires coordinated regulation of ion transport, vacuolar function, and cell wall remodeling, enabling the fungus to maintain cellular homeostasis without relying on extensive morphological transitions.

In contrast, the responses of *C. parapsilosis* to oxygen and pH fluctuations appear to be more closely associated with adhesion and biofilm development. Within sessile communities, progressive oxygen consumption generates steep oxygen gradients that force cells residing in the deeper biofilm layers to reprogram energy metabolism and reduce their proliferative activity [103,104]. These physiological changes may be accompanied by alterations in extracellular matrix production, pseudohyphal morphogenesis, and cell surface composition. Likewise, fluctuations in environmental pH influence cell surface hydrophobicity, adhesion, and the activity of extracellular enzymes, all of which are directly associated with the colonization of medical devices [104,105]. Although the molecular mechanisms underlying these adaptive responses remain less well characterized than in other *Candida* species, the marked variability observed among clinical isolates suggests that oxygen and pH gradients may select for subpopulations with distinct capacities to adhere, persist, and resume growth once environmental conditions become favorable.

In *C. tropicalis*, oxygen availability and environmental pH are closely integrated with the regulation of morphogenesis and biofilm architecture. The deeper layers of biofilms are naturally characterized by hypoxic and nutrient-limited conditions, which promote extensive remodeling of energy metabolism, lipid biosynthesis, and cell wall organization [106]. Likewise, fluctuations in environmental pH modulate filamentation, the activity of hydrolytic enzymes, and the expression of cell surface components involved in adhesion and tissue invasion. The integration of these physicochemical cues with morphological plasticity enables *C. tropicalis* to fine-tune its physiology according to the specific host niche it occupies [107]. However, these adaptive responses are highly isolate-dependent, suggesting that adaptation to hypoxia and pH does not represent a uniform phenotype but rather a source of phenotypic heterogeneity that may influence persistence, immunogenicity, and antifungal tolerance [106].

Adaptation to oxygen and pH gradients is particularly relevant in *C. auris* because of its remarkable ability to colonize human skin, hospital surfaces, and internal host tissues. These environments differ substantially in oxygen availability, humidity, nutrient availability, and pH, requiring an exceptional degree of physiological plasticity to support long-term persistence [108]. Adaptation to oxygen-limited environments is accompanied by metabolic rewiring involving mitochondrial respiration, energy production, sterol biosynthesis, and membrane lipid homeostasis, whereas fluctuations in environmental pH promote coordinated changes in general stress response networks and cell wall architecture. Together, these adaptive responses contribute to biofilm formation, antifungal tolerance, and long-term persistence on abiotic surfaces [96,109]. Consequently, in *C. auris*, adaptation to oxygen and pH fluctuations extends beyond survival within the host and likely contributes to its exceptional capacity to persist and spread in healthcare environments [58].

Finally, in *P. kudriavzevii*, information regarding adaptation to oxygen and pH fluctuations during infection remains limited. Nevertheless, studies conducted in fermentative, food-associated, and industrial environments have demonstrated the ability of this species to maintain growth under acidic conditions and changing oxygen availability [110,111]. Transcriptomic analyses of an acetic acid-tolerant isolate revealed differential expression of genes associated with oxidative phosphorylation, the tricarboxylic acid cycle, glycolysis/gluconeogenesis, and carbon metabolism, suggesting that metabolic reprogramming contributes to the physiological response to organic acid stress [110,112]. Furthermore, studies of aerobic deterioration in fermented feed have associated *P. kudriavzevii* with lactic acid consumption and progressive increases in environmental pH, highlighting its capacity to influence the physicochemical conditions of its surrounding environment under aerobic exposure [112,113]. Together, these observations indicate that *P. kudriavzevii* exhibits considerable metabolic flexibility in response to environmental conditions involving organic acids and oxygen availability. However, because these mechanisms have been investigated predominantly in nonclinical settings, their contribution to adaptation within host-associated microenvironments remains uncertain. Further studies involving clinical isolates and infection-relevant models are therefore required to establish whether comparable metabolic responses contribute to fungal survival and persistence under host-associated fluctuations in oxygen availability and pH.

Collectively, these observations demonstrate that fluctuations in oxygen availability and environmental pH represent shared selective pressures among NAC species, although they do not elicit uniform adaptive responses. Whereas *N. glabratus* relies primarily on maintaining intracellular and energetic homeostasis, *C. parapsilosis* links these environmental cues to adhesion and biofilm development, *C. tropicalis* integrates them with morphological plasticity, *C. auris* exploits them to adapt simultaneously to the host and the hospital environment, and *P. kudriavzevii* appears to capitalize on physiological traits originally acquired in environmental reservoirs. Despite these distinct evolutionary trajectories, all five species converge on a common adaptive outcome: the selection of phenotypes capable of preserving cellular homeostasis, persisting within hostile host niches, and withstanding multiple simultaneous selective pressures, including host immune defenses and antifungal therapy.

### 2.4. Oxidative and Nitrosative Stress

Following adaptation to the physicochemical conditions of the host, NAC species must confront one of the most intense selective pressures encountered during infection: exposure to reactive oxygen species (ROS) and reactive nitrogen species (RNS) generated by cells of the immune system [66,114]. During phagocytosis, neutrophils, macrophages, and other effector cells generate an oxidative burst characterized by the release of superoxide anions, hydrogen peroxide, hydroxyl radicals, nitric oxide, and other reactive intermediates capable of damaging proteins, lipids, nucleic acids, and fungal cell wall components [66,114]. Consequently, the ability to survive within this highly oxidative environment represents a fundamental determinant of fungal persistence and the successful establishment of infection.

From an evolutionary perspective, reactive species not only function as effector mechanisms of innate immunity but also represent important selective forces that drive the adaptation of fungal populations during infection [66,114]. Repeated exposure to ROS and RNS promotes the selection of variants with an enhanced capacity to detoxify reactive compounds, repair molecular damage, reorganize energy metabolism, and remodel the cell wall. These adaptive responses rely on a complex network of antioxidant enzymes, redox homeostasis systems, DNA repair mechanisms, and regulatory circuits that simultaneously coordinate stress responses, metabolism, and virulence [114].

Although NAC species share highly conserved antioxidant mechanisms, including catalases, superoxide dismutases, peroxiredoxins, thioredoxins, and glutathione-dependent systems, accumulating evidence indicates that each species has evolved distinct strategies to cope with oxidative and nitrosative stress according to its evolutionary history, ecological niche, and interaction with the host [27,66]. These differences determine not only the efficiency with which they survive phagocytosis but also their capacity for intracellular persistence, the establishment of chronic infections, and tolerance to other selective pressures encountered during infection.

In *N. glabratus*, resistance to oxidative and nitrosative stress constitutes one of the major determinants of its ability to persist within the host, particularly during intracellular survival in macrophages. Unlike other *Candida* species, *N. glabratus* does not avoid phagocytosis but has instead evolved highly efficient mechanisms to tolerate the oxidative environment generated within the phagolysosome [114,115]. The production of reactive oxygen and nitrogen species by phagocytic cells induces a coordinated antioxidant response that includes the activation of catalases, superoxide dismutases, peroxiredoxins, and glutathione- and thioredoxin-dependent systems, as well as multiple enzymes involved in the repair of oxidative damage [115]. The expression of these systems is regulated primarily by the transcription factors Yap1, Skn7, Msn2, and Msn4, which coordinate the induction of genes involved in redox homeostasis, energy metabolism, and the detoxification of reactive species [100,115,116].

The antioxidant response of *N. glabratus* extends beyond the simple detoxification of free radicals and is integrated with profound physiological reprogramming during host interaction. Exposure to ROS and RNS triggers changes in mitochondrial metabolism, energy production, cell wall remodeling, and cell cycle regulation, thereby promoting survival under prolonged stress conditions [66,114]. Likewise, the close functional cooperation between Yap1 and Skn7 promotes the induction of genes such as *CTA1*, *TRX2*, *TRR1*, and *TSA1*, together with other components of the antioxidant system, ensuring a rapid response to hydrogen peroxide and other oxidants generated by neutrophils and macrophages [115]. In parallel, the response to nitrosative stress depends on partially conserved mechanisms that include the regulation of the flavohemoglobin Yhb1 and other systems responsible for detoxifying nitric oxide and nitrogen-derived reactive species, thereby contributing to intracellular persistence during infection [114,116].

From an evolutionary perspective, resistance to oxidative stress in *N. glabratus* represents not only a defense mechanism against innate immunity but also an adaptive strategy that has favored the selection of populations capable of persisting within phagocytic cells and surviving repeated exposure to ROS and RNS. This remarkable antioxidant capacity explains, at least in part, the exceptional intracellular survival of the species, its persistence during chronic infections, and its close association with therapeutic failure, since many of the regulatory networks involved in the oxidative stress response also participate in antifungal tolerance and adaptation to other selective pressures encountered during infection [100].

In *C. parapsilosis*, the response to oxidative stress appears to be closely integrated with cell surface remodeling and biofilm development. Exposure to hydrogen peroxide modifies the activity of antioxidant enzymes, cell wall morphology, and the repertoire of surface-associated proteins, suggesting that the cell wall actively participates in protection against oxidative damage [117,118]. In sessile cells, several metabolic and stress-response proteins can be relocalized to the cell wall, where they perform additional protective functions, thereby promoting community survival under ROS exposure. This response is particularly relevant during the colonization of catheters and other medical devices, where the extracellular matrix, high cell density, and metabolic heterogeneity of the biofilm can reduce the penetration of oxidants and promote the persistence of more tolerant subpopulations [117,118].

From an evolutionary perspective, oxidative stress may act in *C. parapsilosis* as a selective filter favoring variants capable of more efficiently coordinating antioxidant defenses, cell wall remodeling, and interactions with phagocytic cells [118]. Recent evidence of within-host evolution indicates that derivative isolates can acquire cell wall modifications associated with reduced inflammatory activation and increased survival against neutrophils, supporting the idea that repeated exposure to immune defense mechanisms can progressively reshape the infecting phenotype. However, the specific contribution of RNS and of dedicated nitric oxide detoxification systems remains insufficiently characterized in this species [118].

In *C. tropicalis*, the response to oxidative stress exhibits marked intraspecific heterogeneity. Clinical isolates display substantial differences in their susceptibility to hydrogen peroxide, indicating that antioxidant capacity is not a uniform trait but rather a phenotype shaped by the genetic background and adaptive history of each isolate [119,120,121]. Exposure to oxidants induces changes in the activity of catalases, superoxide dismutases, and glutathione-dependent systems, while also affecting lipid peroxidation, cell wall integrity, and energy metabolism. The variability observed among isolates suggests that, during infection, the oxidative burst generated by phagocytic cells may promote the selection of subpopulations with different capacities to neutralize ROS, repair oxidative damage, and maintain cellular viability [122,123].

Biofilm organization can further enhance this protection by generating gradients of oxygen, nutrients, and metabolic activity that give rise to physiologically differentiated subpopulations [124]. In addition, the elevated catalase activity and other antioxidant mechanisms described in certain isolates may influence polymicrobial interactions by locally reducing hydrogen peroxide concentrations. Collectively, these observations indicate that oxidative adaptation in *C. tropicalis* combines enzymatic defenses, metabolic plasticity, and community organization. Nevertheless, as in *C. parapsilosis*, the specific response to nitrosative stress and its contribution to persistence during infection remain considerably less well understood than the response to ROS [121,125].

In *C. auris*, the interaction with immune cells reveals a particularly complex adaptive strategy. This species can be internalized by macrophages and trigger ROS production, but the extent of phagocytosis and the magnitude of the oxidative response vary among isolates, clades, and cellular states [126]. In addition to tolerating oxidative stress, *C. auris* reduces effective interactions with neutrophils through specific surface characteristics, including distinctive mannosylation patterns that diminish immune recognition and clearance [127,128]. Therefore, its success depends not only on detoxifying ROS once they have been generated but also on limiting the activation of the cellular mechanisms responsible for mounting the oxidative attack. When oxidative exposure occurs, *C. auris* activates antioxidant and repair systems that include peroxiredoxins, catalases, superoxide dismutases, and mechanisms involved in maintaining redox homeostasis [129,130]. The peroxiredoxin Tsa1b plays a particularly important role, as its loss increases susceptibility to dendritic cells and macrophages and reduces disease progression in animal models, thereby directly linking peroxide detoxification with virulence [130]. Likewise, the capacity to withstand oxidative stress may differ between azole-resistant and azole-susceptible isolates, indicating that antifungal resistance may be associated with fitness advantages or trade-offs under oxidative stress conditions [129].

These responses are integrated with other persistence mechanisms, including alterations in lipid metabolism and the formation of large lipid droplets, which have been associated with increased tolerance to diverse environmental stresses, antifungal agents, and host antimicrobial peptides [131]. Thus, in *C. auris*, adaptation to immune-mediated stress combines immune evasion, antioxidant defenses, and physiological reprogramming, generating a phenotype particularly well suited for persistence both within the host and in the hospital environment. Direct characterization of the response to RNS remains comparatively limited and should not be interpreted with the same level of detail as the response to ROS [130,131].

Finally, in *P. kudriavzevii*, information regarding the oxidative and nitrosative stress response during infection remains limited [132]. Available evidence derives primarily from physiological and biotechnological studies, in which exposure to metals, organic acids, heat, and other adverse conditions has been associated with ROS accumulation and the activation of antioxidant systems involving catalase, superoxide dismutase, glutathione, and cellular repair mechanisms. These responses contribute to the maintenance of redox homeostasis and may partially explain the tolerance of this species to multiple environmental stressors [132]. However, studies involving clinical isolates have also provided evidence of specific mechanisms associated with oxidative stress protection. In particular, the presence of an alternative oxidase (AOX) has been demonstrated in *P. kudriavzevii*, and inhibition of this respiratory pathway has been associated with increased intracellular ROS accumulation under oxidative stress conditions [133,134]. Changes in cell wall-associated proteins have also been identified in sessile cells exposed to hydrogen peroxide, suggesting that oxidative stress responses may involve modifications at the fungal cell surface in addition to intracellular antioxidant defenses [135,136]. Nevertheless, the contribution of these mechanisms to survival following phagocytosis and resistance to the oxidative burst generated by neutrophils and macrophages remains insufficiently characterized. Likewise, the mechanisms involved in detoxifying host-derived reactive nitrogen species have not been clearly established. Thus, although the available findings demonstrate that *P. kudriavzevii* possesses physiological mechanisms for coping with oxidative stress, further studies using infection-relevant models are required to determine their contribution to fungal survival and persistence within the host.

All five species share the need to maintain redox homeostasis, but they achieve this objective through different combinations of adaptive strategies. *N. glabratus* is characterized by its remarkable intramacrophage survival and robust antioxidant regulatory networks; *C. parapsilosis* integrates the redox response with cell wall remodeling and biofilm development; *C. tropicalis* exhibits pronounced heterogeneity among clinical isolates; *C. auris* combines immune evasion with highly efficient antioxidant mechanisms; and *P. kudriavzevii* appears to exploit stress tolerance systems that were originally selected in environmental niches. Collectively, these differences suggest that ROS and RNS, together with thermal, nutritional, and physicochemical constraints, act as interconnected host-derived selective pressures that may influence the composition of infecting fungal populations and favor the survival of phenotypes capable of withstanding innate immune defenses (Table 1).

## 3. Cell Wall Remodeling as a Dynamic Host–Pathogen Interface

The cell wall constitutes the first fungal structure to meet the host and, at the same time, one of the primary targets of the selective pressures encountered during infection [137,138]. Far from being a rigid or static structure, the cell wall represents a highly dynamic compartment whose composition and organization continuously change in response to microenvironmental conditions. During colonization and infection progression, *Candida* species must constantly remodel the proportion and distribution of chitin, β-glucans, and mannoproteins to preserve cell integrity in the face of fluctuations in temperature, nutrient availability, pH, oxygen, oxidative stress, and antifungal exposure [66,137].

From an evolutionary perspective, cell wall remodeling represents much more than a structural mechanism of adaptation. Modifications in cell wall architecture simultaneously alter the mechanical properties of the cell, its permeability to external molecules, adhesion to biotic and abiotic surfaces, biofilm formation, and recognition by immune receptors [137]. Consequently, the cell wall functions as a dynamic interface between the fungus and the host, integrating environmental signals with physiological responses and coordinately regulating survival, persistence, and virulence during infection [137].

Although NAC species share the basic organization of the cell wall, consisting of an inner layer rich in chitin and β-glucans covered by an outer layer of mannoproteins, accumulating evidence demonstrates that each species has evolved distinct remodeling strategies that reflect its evolutionary history, ecological niches, and the predominant selective pressures encountered within the host [12,137,139]. These differences determine not only resistance to different types of environmental stress but also the differential exposure of PAMPs, the intensity of immune recognition, the capacity to form biofilms, and susceptibility to antifungal agents [139]. Consequently, cell wall remodeling constitutes one of the principal mechanisms through which *Candida* species adapt their phenotype to the changing conditions encountered within the host.

In *N. glabratus*, cell wall remodeling constitutes an essential adaptive mechanism for maintaining structural integrity and modulating host–pathogen interactions during infection [82,137]. Unlike highly dimorphic species such as *C. albicans*, *N. glabratus* exhibits a predominantly yeast-form mode of growth; consequently, much of its phenotypic plasticity depends on changes in cell wall composition and organization rather than on extensive morphological transitions [82]. Exposure to stress conditions, including nutrient limitation, oxidative stress, pH fluctuations, or antifungal agents, induces coordinated modifications in the content of chitin, β-glucans, and mannoproteins, thereby preserving the mechanical strength of the cell wall and maintaining cellular homeostasis [66,137].

These structural modifications also profoundly influence interactions with the immune system. Reorganization of the outer mannoprotein layer can alter the exposure of β-1,3-glucans and other PAMPs recognized by receptors such as Dectin-1, TLR2, and several C-type lectin receptors expressed on phagocytic cells [139,140]. Consequently, cell wall remodeling not only protects the fungal cell against environmental insults but also simultaneously regulates the intensity of immune recognition, cytokine production, and the efficiency of phagocytosis. In parallel, changes in the expression of adhesins belonging to the *EPA* family, many of which are located within highly variable subtelomeric regions, modify adhesion to epithelial cells, endothelial cells, and abiotic surfaces, thereby promoting colonization of different host niches [141,142].

In *C. parapsilosis*, cell wall remodeling is closely linked to protein glycosylation, adhesion, and biofilm formation. *N*- and *O*-mannosylation pathways contribute to maintaining cell wall organization and regulating the exposure of components recognized by the immune system [143]. Experimental disruption of these pathways alters the content of mannans, glucans, and chitin, compromises cell wall integrity, and modifies the ability of fungal cells to adhere, form pseudohyphae, and interact with phagocytes. Therefore, glycosylation is not merely a biosynthetic process but also a mechanism that organizes the cell surface interface and determines the magnitude of the immune response against this species [143]. This plasticity becomes particularly important during antifungal exposure and within-host evolution. The analysis of sequential isolates recovered during persistent candidemia revealed an increase in mannan content and a reduction in β-glucans, accompanied by increased tolerance to echinocandins, alterations in biofilm formation, and reduced activation of inflammatory responses [118]. These remodeled isolates induced a less pro-inflammatory macrophage polarization and exhibited improved survival against neutrophils, demonstrating that the same cell wall modification can simultaneously provide pharmacological and immunological protection, while also generating adaptive trade-offs, such as increased susceptibility to amphotericin B. Thus, in *C. parapsilosis*, the cell wall functions as a point of convergence between within-host adaptation, therapeutic tolerance, and immune modulation [118].

In *C. tropicalis*, the functional composition of the cell wall differs from that of other species, resulting in a distinct pattern of immune recognition. Protein-linked mannans play a central role in the organization of the outer cell wall layer, the maintenance of cellular integrity, and interactions with host cells [144]. Disruption of genes involved in *N*- and *O*-mannosylation alters the distribution of cell wall polysaccharides, increases β-glucan exposure, and modifies cytokine production by immune cells. These findings demonstrate that the mannan layer acts as a dynamic regulator of PAMP accessibility rather than simply serving as a structural coating [144,145]. Furthermore, recognition of *C. tropicalis* depends on the differential contribution of receptors such as Dectin-1, TLR2, TLR4, and the mannose receptor, and varies according to the state of the cell wall and the type of immune cell involved [145]. Differences in β-glucan exposure and mannan structure can result in distinct patterns of phagocytosis and cytokine secretion, demonstrating that relatively small modifications at the cell surface have broad consequences for the host immune response. This variability may also occur among clinical isolates, making cell wall remodeling an additional source of phenotypic heterogeneity with potential consequences for virulence, persistence, and antifungal susceptibility [145,146].

In *C. auris*, the cell wall constitutes a particularly important determinant of immune evasion, persistence, and antifungal resistance. Although it retains the general organization consisting of an inner layer of chitin and β-glucans covered by an outer layer rich in mannans, it exhibits distinctive structural and glycosylation features that contribute to its unique clinical behavior [127]. Cell wall mannosylation reduces effective interactions with neutrophils: disruption of genes such as *PMR1* or *VAN1* decreases the abundance of outer mannans, exposes immunostimulatory ligands, and increases neutrophil contact, phagocytosis, and neutrophil-mediated killing. In this way, the mannan architecture of *C. auris* actively functions as a mechanism of immune masking [127]. β-Glucan exposure is also highly dynamic in this species. Host-relevant conditions, including the presence of lactate, hypoxia, acidic pH, or sublethal concentrations of fluconazole, modify the amount of β-glucan exposed on the cell surface [108]. Reduced β-glucan exposure decreases macrophage recognition and phagocytosis and has been associated with increased virulence in experimental models, whereas acidic pH may produce the opposite effect. These findings demonstrate that *C. auris* integrates metabolic, physicochemical, and therapeutic signals to regulate the immunological visibility of its cell wall [108].

Antifungal-induced cell wall remodeling adds another level of complexity. As observed in other *Candida* species, inhibition of β-1,3-glucan synthesis can trigger compensatory responses that increase chitin content and reorganize the polysaccharide network, thereby temporarily preserving cell wall integrity and promoting antifungal tolerance [24,147]. However, the magnitude of these responses varies among species and clinical isolates, contributing to the clinical heterogeneity observed in *C. auris*. Therefore, its cell wall not only provides protection against the external environment but also functions as a regulatory platform linking antifungal resistance, skin colonization, biofilm formation, and immune evasion [147].

Finally, in *P. kudriavzevii*, knowledge regarding cell wall remodeling during infection remains more limited. Nevertheless, it has been demonstrated that its cell wall mannans possess distinctive immunomodulatory properties: they can be recognized through TLR2 and the MyD88 signaling pathway, induce dendritic cell apoptosis, and promote a Th17-type immune polarization [64,135]. These findings indicate that the surface of *C. krusei* is not merely a mechanical barrier but rather a structure capable of qualitatively shaping the host immune response [135]. Alterations in cell wall composition have also been reported in amphotericin B-resistant isolates, including variations in glucans, chitin, and other components that affect PAMP exposure and immune recognition. However, it remains unclear to what extent these alterations represent regulated responses during infection, adaptations selected by antifungal exposure, or traits derived from the species’ remarkable ecological tolerance [110,111]. Therefore, in *P. kudriavzevii*, the relationship between cell wall plasticity and pathogenicity should still be regarded as a relatively unexplored area, in which it is particularly important to distinguish evidence obtained from clinical isolates from that derived from industrial or environmental conditions [135].

Importantly, the consequences of cell wall remodeling during host–pathogen interactions are influenced not only by fungal species-specific characteristics but also by the immunological status of the host [139]. In immunocompetent individuals, recognition of cell wall-associated PAMPs by pattern recognition receptors expressed on neutrophils, macrophages, and other immune cells contributes to fungal clearance through phagocytosis, oxidative and nonoxidative antimicrobial mechanisms, and inflammatory signaling. In contrast, neutropenic patients experience a substantial reduction in neutrophil-mediated antifungal defenses, potentially modifying the selective pressures encountered by fungal populations during infection [148,149]. Under these conditions, the relative contribution of other immune effector cells and tissue-associated defense mechanisms may become particularly important. Nevertheless, the absence or reduction in specific immune cell populations does not eliminate immune-mediated selective pressures, and the consequences of fungal cell wall remodeling for immune recognition and survival may therefore differ according to the immunological background of the patient [139,149].

Beyond host immune status, the anatomical site of infection further determines the nature and intensity of host-derived selective pressures. At mucosal surfaces, fungal cells interact with epithelial barriers, antimicrobial peptides, resident microbiota, and locally coordinated immune responses, including IL-17-associated protective mechanisms [139,150,151]. During bloodstream infection, circulating fungal cells encounter complement proteins, phagocytic cells, and other systemic immune effectors, whereas tissue-invasive infections involve additional interactions with recruited inflammatory cells and microenvironments characterized by variable oxygen availability, nutrient restriction, and tissue damage. In device-associated infections, biofilm formation and extracellular matrix accumulation create spatially heterogeneous environments that may limit immune cell access and reduce antifungal susceptibility [148,152]. Consequently, the biological significance of cell wall remodeling extends beyond its structural role, as changes in the exposure of immunologically active components may have different consequences depending on the immune effector mechanisms operating within each host niche. However, the extent to which these context-dependent interactions contribute to the persistence of individual NAC species remains incompletely understood [139].

Although all five NAC species share the capacity to dynamically reorganize their cell wall, they employ distinct strategies that influence their interactions with the host. *N. glabratus* combines adhesin variation with modulation of PAMP exposure; *C. parapsilosis* links cell wall remodeling to biofilm formation, altered echinocandin susceptibility, and modulation of immune responses; *C. tropicalis* relies on mannosylation-dependent mechanisms that influence β-glucan exposure; *C. auris* exhibits distinctive mannan structures and dynamic changes in β-glucan exposure that contribute to immune evasion and persistence; and *P. kudriavzevii* displays mannans with distinctive immunomodulatory properties. Importantly, as discussed above, the consequences of these adaptations are also shaped by the immunological status of the host and the anatomical microenvironment in which infection occurs. Thus, cell wall remodeling should be understood as a context-dependent adaptive process in which species-specific structural characteristics interact with environmental, immunological, and therapeutic pressures to influence fungal recognition, survival, and persistence (Table 2).

Collectively, the adaptive responses described throughout this review demonstrate that NAC species withstand host-derived selective pressures through coordinated modifications in their physiology, metabolism, and cellular architecture. While these mechanisms contribute to fungal survival under diverse environmental and immunological conditions, they may also facilitate the development of phenotypic states that enable fungal populations to withstand antifungal exposure without necessarily acquiring stable genetic resistance [153,154]. In this context, antifungal tolerance and persistence represent distinct survival phenotypes that may contribute to prolonged infection and provide opportunities for the subsequent selection of resistant variants [153]. Understanding how these phenotypes emerge and are maintained under the combined influence of host-derived and therapeutic pressures is therefore essential for elucidating the mechanisms underlying persistent NAC infections and the challenges associated with their eradication.

## 4. Emergence of Persistence and Antifungal Tolerance

The epidemiology of microbial infections is rapidly changing due to stressors such as climate change, increases in antimicrobial resistance, and high population migration activity [155]. Invasive fungal infections have become an increasingly important cause of severe healthcare-associated infections, with mortality rates ranging from 30% to 40% among critically ill patients. Approximately 80% of invasive fungal infections occurring in intensive care units (ICUs) are caused by *Candida* species [156].

*Candida* bloodstream infection (candidemia) has been considered a major cause of morbidity and mortality in the hospital setting, and changes are currently occurring in the epidemiology of *Candida* infections [157,158]. In this regard, the incidence of invasive candidiasis varies in different countries, ranging between 3.4% and 5.79%, while the crude mortality rate from candidemia during hospitalization has reached up to 72.2%. Contrastingly, some reports from North America and Europe show that the mortality rate of candidemia exceeds 50% [158,159]. Unfortunately, a universal candidemia rate, a methodology for calculating the rate, and established criteria for an incidence denominator are currently unavailable; therefore, comparing infection rates between regions is a complex task [160,161]. Despite these methodological limitations, epidemiological studies consistently indicate that the incidence of candidemia has increased worldwide over the last decade and varies between 1.5/100,000 and 33.8/100,000 individuals, depending on the country and clinical setting, likely due to the high complexity of surgical procedures, the presence of patient groups at high risk of infection, and changes in patient demographics [162,163].

It should be noted that invasive candidiasis encompasses a broad spectrum of disease manifestations beyond candidemia. However, because bloodstream infections are more readily recognized and microbiologically confirmed than deep-seated invasive candidiasis, most epidemiological studies have focused primarily on candidemia [155,160]. Furthermore, the epidemiology of candidemia varies according to geographic region, patient health status, and hospital system. Thus, risk factors associated with candidemia include the use of immunosuppressive agents such as corticosteroids and broad-spectrum antibiotics, and the increased use of invasive procedures in medical practice, especially in patients with hematological diseases, neutropenia, and diabetes mellitus [164,165].

An important limitation of studies assessing the epidemiology of invasive candidiasis and antifungal susceptibility is the reliance on blood culture-positive cases. Because blood cultures have suboptimal sensitivity, only a fraction of invasive candidiasis episodes are captured, resulting in an incomplete representation of the overall disease burden [166]. Moreover, species identification and antifungal susceptibility testing are not consistently performed for all culture-positive isolates, potentially introducing additional bias. Nevertheless, in several countries and healthcare settings where these procedures are routinely implemented or mandated, the available data are considered sufficiently representative to describe species distribution and antifungal susceptibility trends [166]. Despite these limitations, epidemiological studies consistently demonstrate a steady increase in the prevalence of NAC species worldwide.

This epidemiological shift has been associated, in part, with the selective pressure imposed by the widespread use of antifungal agents, since many of these species exhibit reduced susceptibility or intrinsic resistance to commonly used antifungals [166]. Although this epidemiological shift has traditionally been attributed to the reduced susceptibility or intrinsic resistance of NAC species to antifungal agents, accumulating evidence indicates that antifungal resistance alone cannot fully explain treatment failure. Clinical isolates classified as susceptible according to conventional minimum inhibitory concentration (MIC) breakpoints may still survive prolonged antifungal exposure, leading to persistent infections, recurrent disease, and poor clinical outcomes. These observations have led to growing interest in non-genetic survival strategies, particularly antifungal tolerance and persistence, which are now recognized as important contributors to therapeutic failure. Consequently, the current paradigm has shifted from viewing antifungal resistance as the sole determinant of therapeutic failure to recognizing antifungal tolerance and persistence as distinct, yet complementary, phenotypic strategies that promote fungal survival despite antifungal exposure.

### Defining Antifungal Tolerance and Persistence

Antifungal tolerance is defined as the ability of an otherwise drug-susceptible fungal population to survive and grow slowly at antifungal concentrations exceeding the MIC [167]. Importantly, antifungal tolerance should not be confused with antifungal resistance. Whereas resistance is reflected by an elevated MIC resulting from heritable genetic changes, tolerant isolates often display MIC values within the susceptible range but retain the ability to survive prolonged antifungal exposure [168]. Consequently, MIC values alone do not always predict the fungicidal activity of an antifungal agent or its clinical efficacy. The fungicidal or fungistatic activity of antifungal agents depends on both the drug class and the fungal species. For example, azoles are generally fungistatic against most *Candida* species but fungicidal against *Aspergillus* spp., whereas echinocandins exhibit fungicidal activity against most *Candida* species while remaining primarily fungistatic against *Aspergillus* spp. [168]. Importantly, antifungal tolerance is also drug- and species-dependent. For instance, tolerance to echinocandins in *N. glabratus*, despite these drugs being considered fungicidal against this species, allows a small subpopulation of cells (approximately 10^−4^–10^−5^ of the total population) to survive across a broad range of drug concentrations [168,169].

Although closely related, antifungal tolerance and persistence represent distinct phenotypic survival strategies. Tolerance generally involves the survival or slow growth of a relatively larger fraction of the fungal population above the MIC, whereas persistence is restricted to a rare subpopulation of cells that withstand prolonged fungicidal exposure without acquiring heritable resistance [170]. Persister cells have been studied primarily in *Candida* biofilms, where they constitute a very small fraction of the total population yet survive exposure to high concentrations of amphotericin B, echinocandins, and other fungicidal antifungal agents. Following drug withdrawal, these surviving cells can regenerate a fully susceptible population, thereby contributing to recurrent infections and therapeutic failure [170,171]. The clinical relevance of persistence lies in its potential to promote chronic and recurrent infections despite appropriate antifungal therapy. Although persister cells do not harbor stable resistance mutations, their prolonged survival under antifungal pressure increases the opportunity for adaptive evolution and may facilitate the subsequent emergence of genetically resistant populations [168].

From a diagnostic perspective, distinguishing antifungal resistance, tolerance, and persistence remains challenging because conventional antifungal susceptibility testing primarily relies on minimum inhibitory concentration (MIC) determination. Although standardized CLSI and EUCAST methodologies provide essential information for identifying reduced antifungal susceptibility, MIC measurements do not fully characterize the heterogeneous responses of fungal populations during prolonged drug exposure. Consequently, isolates exhibiting similar MIC values may differ substantially in their capacity to maintain residual growth or survive exposure to antifungal concentrations exceeding the MIC. This limitation has encouraged the development of complementary experimental approaches aimed at characterizing antifungal tolerance and persistence beyond conventional susceptibility endpoints [170].

Several methods have been explored to quantify these phenotypes, including measurements of residual growth above the MIC, assessments of growth dynamics during prolonged antifungal exposure, and time-kill assays that evaluate the survival of fungal populations over time. In particular, the fraction of growth above the MIC (FoG) has been proposed as a quantitative measure of antifungal tolerance under specific experimental conditions, whereas survival-based assays may help identify rare persister subpopulations capable of withstanding prolonged exposure to fungicidal agents [170]. However, the interpretation of these measurements depends on the antifungal compound, fungal species, experimental conditions, and duration of drug exposure. Furthermore, tolerance and persistence are not interchangeable phenotypes, and the detection of residual growth above the MIC does not necessarily demonstrate the presence of persister cells. Despite their potential value, these approaches have not yet been standardized or clinically validated for routine implementation across NAC species, and their ability to predict therapeutic outcomes remains incompletely established [153,168].

The phenotypic plasticity underlying antifungal tolerance and persistence is supported by a variety of adaptive cellular responses, including stress-response signaling, cell wall remodeling, metabolic reprogramming, multicellular aggregation, and genome plasticity. These mechanisms differ among *Candida* species but collectively enhance fungal survival under prolonged antifungal exposure.

*Candida* species can adapt to host- and drug-induced stress through morphological and cellular remodeling. Although dimorphism has traditionally been associated with the yeast-to-hypha transition in *C. albicans*, other adaptive morphologies, including multicellular aggregation, have increasingly been recognized as survival strategies during antifungal exposure. In *C. auris*, two types of multicellular aggregates have been described: one mediated by ALS-family adhesins and another resulting from defects in cytokinesis, in which daughter cells fail to detach from the mother cell after budding [172]. Under high echinocandin concentrations, some clinical isolates of *Candida* spp. exhibit discontinuous growth, also known as paradoxical growth, accompanied by the formation of multicellular aggregates [173]. In *C. auris*, echinocandin-induced aggregates are primarily associated with defective cell separation, whereas in *C. albicans*, aggregation appears to depend mainly on adhesin-mediated interactions [172,174]. Importantly, aggregate-forming strains of *C. auris* and *N. glabratus* have been associated with enhanced virulence and increased resistance to immune-mediated killing [175,176]. Thus, multicellular aggregation may represent an adaptive phenotype that links cell wall remodeling, antifungal tolerance, immune evasion, and pathogenicity.

In *N. glabratus*, antifungal tolerance appears to result from the coordinated activation of stress-response pathways, including calcineurin signaling, enhanced drug efflux, mitochondrial adaptation, and extensive genome plasticity. These mechanisms promote fungal survival during prolonged antifungal exposure and may facilitate the subsequent evolution of stable antifungal resistance [177]. Compared with *N. glabratus* and *C. albicans*, the molecular basis of antifungal tolerance in *C. parapsilosis* remains considerably less understood. Current evidence suggests that tolerance is associated with alterations in *FKS1*, cellular stress-response pathways, and biofilm-associated survival; however, the regulatory networks underlying this phenotype require further investigation [178]. In *C. tropicalis*, caspofungin tolerance has been linked to the modulation of cell wall integrity and cell division pathways, particularly through increased chitin synthesis and downregulation of genes involved in cell separation. Consistently, overexpression of the cell division-related transcription factor *ACE2* suppresses caspofungin tolerance in *C. tropicalis* by delaying the formation of multicellular aggregates [167].

Unlike *N. glabratus*, in which calcineurin signaling, mitochondrial adaptation, and genome plasticity have been directly implicated in antifungal tolerance, the molecular basis of tolerance in *P. kudriavzevii* remains largely unexplored [179]. Current knowledge is mostly limited to intrinsic azole resistance mechanisms, including reduced Erg11 susceptibility and increased efflux pump activity, whereas the contribution of stress-response pathways to tolerance and persistence remains poorly defined. This limited understanding highlights an important knowledge gap and underscores the need for future studies aimed at identifying the molecular pathways that allow *P. kudriavzevii* to survive prolonged antifungal exposure independently of classical resistance mechanisms. Although persister cells do not harbor stable resistance mutations, their prolonged survival under antifungal pressure increases the opportunity for adaptive evolution and may facilitate the subsequent emergence of genetically resistant populations (Table 3).

## 5. Clinical Implications: Therapeutic Failure and Disease Recurrence

The recognition of antifungal tolerance and persistence has important clinical implications, as these phenotypic survival strategies may compromise therapeutic efficacy even in isolates classified as susceptible according to current antifungal susceptibility testing. Current clinical management of invasive candidiasis relies largely on minimum inhibitory concentration (MIC)-based susceptibility testing to guide therapeutic decisions. However, accumulating evidence indicates that MIC values alone fail to capture adaptive phenotypes such as antifungal tolerance and persistence, which may contribute to therapeutic failure, persistent candidemia, and recurrent infections despite isolates remaining susceptible according to current CLSI or EUCAST breakpoints [170].

Supporting this concept, Rosenberg et al. demonstrated that, in a cohort of patients with persistent candidemia, isolates exhibiting high antifungal tolerance were associated with prolonged bloodstream infection despite remaining susceptible according to conventional MIC testing [170].

From a clinical management perspective, these findings highlight the importance of considering antifungal tolerance and persistence when infections fail to resolve despite treatment with agents to which the corresponding isolates remain susceptible in vitro [153,170]. Nevertheless, persistent or recurrent candidemia is multifactorial and may also reflect inadequate source control, infected intravascular devices, biofilm-associated infections, insufficient antifungal exposure, or underlying host conditions [8]. Therefore, the interpretation of persistent infection requires integrating microbiological findings with the patient’s clinical evolution, reassessment of the infectious focus, and evaluation of antifungal susceptibility. Although experimental characterization of tolerant or persistent subpopulations may eventually contribute to identifying patients at increased risk of therapeutic failure, current evidence does not support modifying antifungal selection, dosing, or treatment duration solely on the basis of these phenotypes. Further clinical studies are required to establish whether their detection provides prognostic information beyond conventional susceptibility testing and established clinical risk factors [153].

Beyond their immediate impact on therapeutic efficacy, tolerant and persistent subpopulations may also promote fungal adaptation during prolonged antifungal exposure. Their survival increases the opportunity for adaptive evolution, thereby facilitating the subsequent emergence of stable resistance mutations. Collectively, these observations suggest that antifungal tolerance and persistence may contribute to therapeutic failure and disease recurrence, highlighting their potential clinical relevance while emphasizing the need for further studies to establish their impact on treatment outcomes [168,177].

Accordingly, future research should investigate whether the assessment of antifungal tolerance, persistence, and other adaptive phenotypes could complement conventional MIC-based susceptibility testing and provide additional information for predicting therapeutic outcomes and understanding disease recurrence. However, further clinical studies and methodological standardization are needed to establish the diagnostic and prognostic value of these approaches before their incorporation into routine clinical practice can be considered.

## 6. Future Perspectives

Despite substantial advances in understanding the mechanisms that enable *Candida* species to survive within the host, important gaps remain in our knowledge of how non-albicans *Candida* species integrate the multiple environmental and immunological challenges encountered during infection. Most available studies have examined individual stressors under controlled experimental conditions, whereas fungal cells within the host are simultaneously exposed to fluctuations in temperature, nutrient and micronutrient availability, oxygen tension, pH, oxidative and nitrosative stress, immune effectors, and antifungal drugs. Future studies should therefore move beyond single-stressor experimental designs toward models that reproduce the combined and dynamic nature of host microenvironments. Such approaches will be essential to determine whether adaptive responses identified under isolated conditions remain relevant when multiple pressures act simultaneously and to establish how these responses influence fungal fitness during infection.

An additional challenge is the marked imbalance in the amount and depth of information available across NAC species. While several adaptive mechanisms have been extensively characterized in *N. glabratus* and, increasingly, in *C. auris*, our understanding of comparable processes in *C. parapsilosis, C. tropicalis*, and particularly *P. kudriavzevii* remains more fragmented. Moreover, species-level comparisons may obscure substantial variability among clinical isolates. Expanding comparative analyses to include isolates from different anatomical sites, geographic origins, clinical backgrounds, and susceptibility profiles will therefore be important for distinguishing conserved species-level responses from strain-specific adaptive phenotypes. This distinction may be particularly relevant for understanding why isolates belonging to the same species can differ in their ability to colonize particular niches, withstand immune-mediated stress, form biofilms, or survive antifungal exposure.

Future research should also address more directly the mechanisms connecting environmental adaptation with persistence and antifungal tolerance. Although metabolic reprogramming, stress responses, redox homeostasis, and cell wall remodeling can promote survival under adverse conditions, the extent to which these processes directly generate or stabilize persistent and tolerant subpopulations remains incompletely understood. In particular, cell wall remodeling deserves further investigation as a potential integrative hub linking environmental sensing with immune recognition and antifungal susceptibility.

Ultimately, understanding NAC pathogenicity requires moving beyond the analysis of individual virulence factors or isolated stress responses toward an integrated view of fungal adaptation within the host. The evidence discussed in this review indicates that clinically relevant NAC species can face common host-derived challenges through distinct physiological strategies while converging on phenotypes that preserve cellular fitness and promote survival and persistence. Defining both the conserved principles and species-specific mechanisms underlying these responses may reveal previously overlooked vulnerabilities and provide a foundation for therapeutic strategies aimed not only at inhibiting fungal growth but also at disrupting the adaptive processes that enable fungal populations to persist despite immune and antifungal pressure.

## Figures and Tables

**Figure 1:**
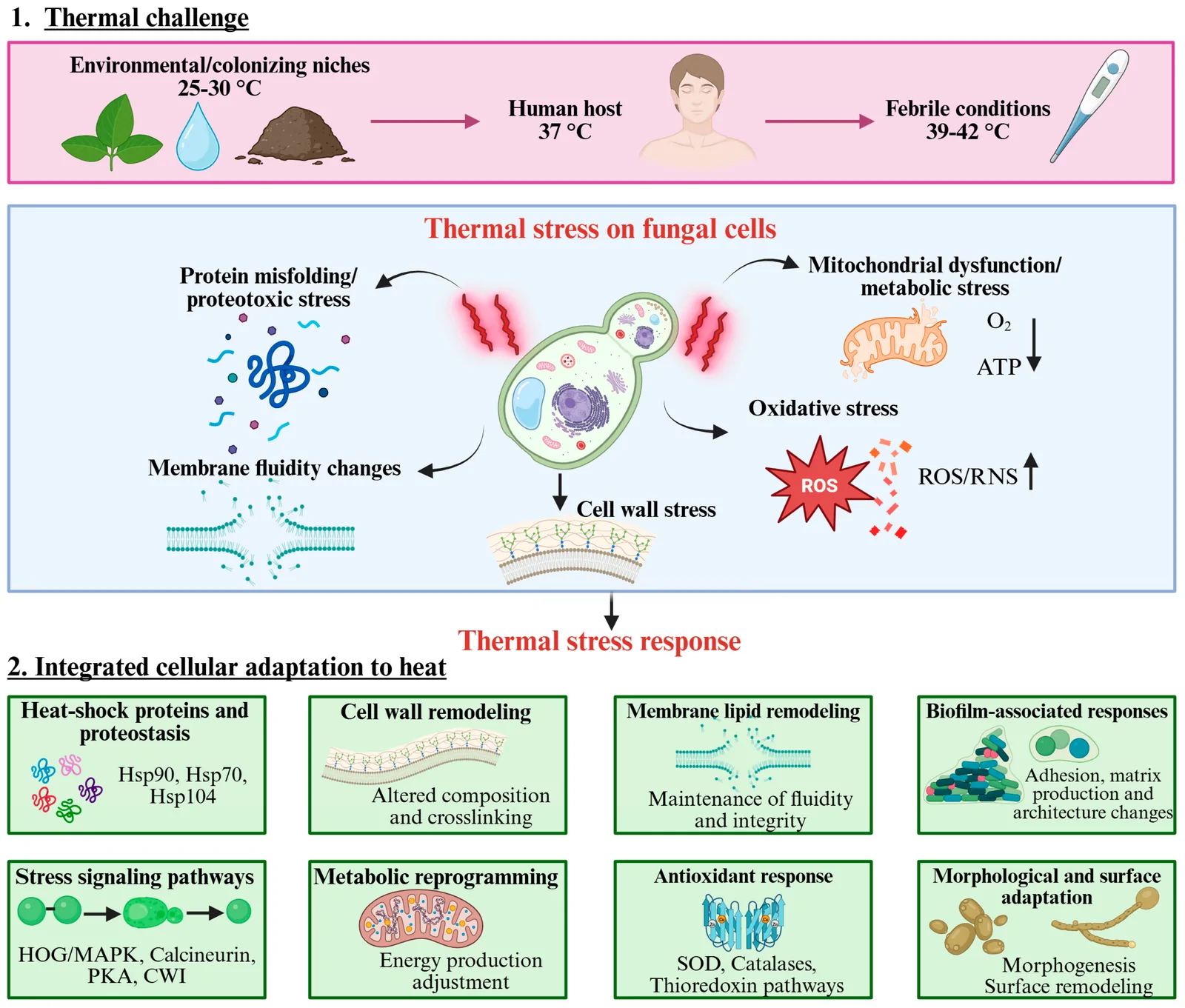
Integrated cellular responses to thermal stress in non-*albicans* *Candida* species. During the transition from environmental or colonizing niches (approximately 25–30 °C) to the human host (37 °C), fungal cells encounter increasing thermal challenges, particularly under febrile conditions (approximately 39–40 °C) or experimental high-temperature stress conditions (up to 42 °C), which can affect multiple cellular processes. (1) Thermal challenge. Elevated temperatures can promote protein misfolding and proteotoxic stress, alter membrane fluidity, compromise cell wall homeostasis, induce mitochondrial and metabolic dysfunction, and increase oxidative stress through the accumulation of reactive oxygen and nitrogen species (ROS/RNS). (2) Integrated cellular adaptation to heat. To counteract these alterations, non-*albicans* *Candida* species activate interconnected adaptive mechanisms, including heat-shock proteins and proteostasis networks, stress-signaling pathways such as HOG/MAPK, calcineurin, PKA, and the cell wall integrity (CWI) pathway, as well as cell wall and membrane lipid remodeling, metabolic reprogramming, antioxidant defenses, biofilm-associated responses, and morphological and surface adaptations. The coordinated activation of these processes contributes to the maintenance of cellular homeostasis and enables fungal cells to withstand the thermal conditions encountered during host colonization and infection.

**Table 1:** Physiological and metabolic responses to host-derived selective pressures across NAC species.

Environmental/Host Selective Pressure	Pathogen	Key Sensors, Pathways and Mediators	Adaptive Physiological and Metabolic Strategy	Impact on Host Colonization and Persistence	References
Thermal Stress (37 °C: body temperature; 39–40 °C: fever; up to 42 °C: experimental heat stress)	*N. glabratus*	HOG (Sho1 branch), CWI pathways; Msn2/Msn4; CgSte11 (MAPKKK); Hsp90	Redistribution of energetic resources; downregulation of mitochondrial respiration genes; cross-protection to oxidative stress and organic acids	Growth at 42 °C; multi-stress cross-tolerance; intramacrophage resilience	[42,43,44,45,47]
	*C. parapsilosis*	Temperature-dependent cell surface signaling	Modulation of cell surface hydrophobicity and surface free energy; uncoupling of initial adhesion from biofilm maturation	Device colonization across broad temperature ranges; nosocomial surface persistence	[34,49,50]
	*C. tropicalis*	Hsp networks, CWI pathway	Interplay between thermal signaling, filamentation, and biofilm matrix restructuring	Niche colonization; adaptive differentiation during febrile episodes	[36,53,54]
	*C. auris*	Hsp90; HOG (Hog1), calcineurin, PKA, and CWI signaling	High thermotolerance (up to 42 °C); coordinated cell wall and biofilm remodeling	Overcoming mammalian thermal barrier; skin colonization and hospital environmental spread	[56,57,58,59,60]
	*P. kudriavzevii*	Heat shock proteins; antioxidant networks; central carbon flux rewiring	Growth at 40–45 °C and metabolic adjustments under thermal stress, primarily characterized in environmental and biotechnological isolates	Thermotolerance demonstrated under environmental and laboratory conditions; potential contribution to survival at mammalian temperatures remains unconfirmed during infection	[58,62,63]
pH and Hypoxia Fluctuations	*N. glabratus*	Sterol/lipid remodeling pathways; vacuolar ion transporters	Maintenance of ATP and energy homeostasis during oxygen restriction; membrane stabilization without morphological transition	Survival within hypoxic, highly acidic phagolysosomes	[100,101,102]
	*C. parapsilosis*	Biofilm metabolic stratification networks	Metabolic downregulation in deep biofilm layers; pH-dependent modulation of surface hydrophobicity	Catheter persistence and tolerance to deep-layer hypoxia	[103,104,105]
	*C. tropicalis*	Morphogenetic and metabolic circuits	Hypoxia-driven matrix synthesis; pH-dependent regulation of filamentation and secreted hydrolases	Deep tissue penetration and adaptation within dense biofilms	[106,107]
	*C. auris*	General stress response circuits; sterol biosynthesis	Dynamic respiratory adjustments under hypoxia; survival across wide pH gradients	Persistence on diverse abiotic and biotic surfaces	[96,108,109]
	*P. kudriavzevii*	Respiratory-to-fermentative metabolic switching	Acid tolerance and metabolic reprogramming in response to organic acid stress; environmental pH changes associated with aerobic growth	Acid tolerance and metabolic flexibility demonstrated in nonclinical settings; potential relevance to host-associated pH and oxygen fluctuations remains to be established	[110,111,112,113]
Oxidative and Nitrosative Stress	*N. glabratus*	Yap1, Skn7, Msn2, Msn4; Cta1, Trx2, Trr1, Tsa1; Flavohemoglobin (Yhb1)	Coordinated antioxidant enzyme activation; repair systems; neutralization of reactive nitrogen species (RNS)	Intracellular survival during phagocytic oxidative burst; evasion of immune clearance	[100,114,115,116]
	*C. parapsilosis*	Cell wall moonlighting antioxidant proteins; biofilm protective matrix	Relocalization of antioxidant enzymes to the cell wall; suppression of pro-inflammatory macrophage activation	Attenuation of neutrophil killing during device-associated infections	[118]
	*C. tropicalis*	Catalases, superoxide dismutases (SODs), glutathione system	High intraspecific variation in ROS neutralization; catalase-mediated protective microenvironments in biofilms	Interspecies biofilm survival and strain-dependent resistance to phagocyte killing	[119,120,121]
	*C. auris*	Peroxiredoxin Tsa1b; outer mannosylation masking machinery; giant lipid droplets	Active immune masking to avoid neutrophil attack; robust peroxide detoxification; lipid droplet accumulation	Virulence maintenance; reduction in immune recognition and phagocytic clearance	[126,127,128,129,130]
	*P. kudriavzevii*	Catalase, SOD, and glutathione systems	Antioxidant responses and redox homeostasis characterized mainly under environmental or industrial stress conditions	Environmental oxidative stress tolerance; contribution to resistance against host immune-mediated oxidative killing remains unverified	[132,133,134]

**Table 2:** Cell wall remodeling, immune interface dynamics, and host–pathogen interactions.

Species	Cell Wall Remodeling Characteristics	Key Biosynthetic and Regulatory Pathways	Impact on Immune Recognition (PAMP Exposure and Receptors)	Impact on Adhesion, Virulence and Antifungal Susceptibility	References
*N. glabratus*	Yeast-restricted remodeling; compensatory chitin/*β*-glucan balance; dynamic changes in outer mannoprotein architecture	Subtelomeric *EPA* adhesins; cell wall integrity (CWI) pathway; glycosylation enzymes	Modulation of exposed *β*-(1,3)-glucans; differential activation of Dectin-1 and TLR2	Dynamic adhesion to biotic/abiotic surfaces; immune avoidance inside macrophages; echinocandin tolerance	[66,82,137,139,140]
*C. parapsilosis*	Marked shifts in mannan/*β*-glucan ratios during systemic infection; cell wall protein relocalization	*N*- and *O*-linked protein mannosylation circuits	Mannan shielding masks immunostimulatory *β*-glucans; promotes anti-inflammatory macrophage polarization	Enhanced survival against neutrophil killing; concurrent echinocandin tolerance with amphotericin B hypersensitivity trade-offs	[118,143]
*C. tropicalis*	Mannan-dependent regulation of inner wall accessibility; strain-specific outer polysaccharide layer depth	*N*- and *O*-mannosylation cascades; cell separation machinery	Variable *β*-glucan exposure drives differential engagement with Dectin-1, TLR2, TLR4, and Mannose Receptor (MR)	Altered cytokine profiles; strain-specific fitness and persistence in mucosal niches	[144,145,146]
*C. auris*	Unique mannan architecture; metabolic/physicochemical regulation of *β*-glucan exposure (lactate, pH, hypoxia)	*PMR1*, *VAN1*; compensatory chitin synthesis during *β*-glucan synthase inhibition	Outer mannan layer actively prevents neutrophil activation and NET formation; microenvironment-dependent immune masking	Enhanced skin colonization; marked immune evasion; elevated basal and drug-induced echinocandin tolerance	[108,127]
*P. kudriavzevii*	Distinctive mannan immunomodulatory properties; surface alterations in drug-resistant isolates	Distinct mannosyltransferases; cell surface stress-response proteome	Mannans trigger TLR2/MyD88 signaling, induce dendritic cell apoptosis, and drive Th17 immune polarization	Qualitatively directs adaptive immune responses; potential contribution to persistence	[64,110,111,135]

**Table 3:** Comparison of antifungal resistance, tolerance, and persistence in non-*albicans Candida* species.

Parameter	Antifungal Resistance	Antifungal Tolerance	Antifungal Persistence
Definition	Heritable ability of a fungal cell/population to grow at elevated antifungal concentrations	Non-heritable ability of an otherwise susceptible population to survive and grow slowly at concentrations above the MIC.	Survival of a rare, specialized subpopulation under fungicidal drug exposure without genetic changes.
Clinical Laboratory Metric	Increased Minimum Inhibitory Concentration (MIC) above established CLSI/EUCAST clinical breakpoints.	Supra-MIC slow growth/trailing growth; disk diffusion clearance zones; tolerance metrics (fraction of growing population).	Biphasic killing curves; time-kill assay survival plateaus; persister cell frequency counts.
Genetic Basis	Stable, heritable mutations, gene amplifications, or stable chromosomal aneuploidies.	Non-genetic/epigenetic adaptation; transient gene expression shifts; reversible segmental aneuploidies.	Phenotypic dormancy; specialized physiological/dormant state; reversible upon drug removal.
Fraction of Population Involved	Entire population (or majority clonal fraction).	Intermediate to large subpopulation (10^−1^ to 10^−3^).	Extremely small minority subpopulation (10^−4^ to 10^−6^).
Primary Mechanisms in NAC Species	Point mutations in *ERG11* (e.g., Y132F in *C. parapsilosis*, *C. auris*).	Calcineurin and Hsp90 stress signaling (*N. glabratus*, *C. auris*).	Biofilm-embedded dormant cells protected by extracellular matrix.
	*FKS1* hot-spot mutations (*N. glabratus*, *C. krusei*, *C. auris*).	Compensatory chitin upregulation during echinocandin exposure.	Metabolic shutdown and mitochondrial downregulation.
	*PDR1*/*TAC1B* gain-of-function mutations driving efflux overexpression.	*ACE2* downregulation and multicellular aggregation (*C. tropicalis*, *C. auris*). Reversible aneuploidies (*C. parapsilosis*).	Giant lipid droplet formation (*C. auris*) Relocalization of stress proteins to the cell wall.
Clinical Consequences	Direct microbiological and therapeutic failure; requirement for alternative antifungal classes.	Delayed fungal clearance; persistent candidemia despite “susceptible” MIC; risk of breakthrough infection.	Recurrent infection/disease relapse after treatment cessation; evolutionary reservoir for selecting stable resistance mutations.
Key References	[94,169,178]	[153,167,170,177,178]	[121,153,154,171]

## Data Availability

The original contributions presented in the study are included in the article, further inquiries can be directed to the corresponding author.
